# CoSn_3_ Intermetallic Nanoparticles for Electronic Packaging

**DOI:** 10.3390/nano12224083

**Published:** 2022-11-20

**Authors:** Jintao Wang, Ziwen Lv, Luobin Zhang, Fangcheng Duan, Weiwei Zhang, Hongtao Chen

**Affiliations:** 1 Department of Materials Science and Engineering, Harbin Institute of Technology (Shenzhen), Shenzhen 518055, China; 2 State Key Lab of Advanced Solder Ang Joining, Harbin Institute of Technology, Harbin 150001, China; 3 Sauvage Laboratory for Smart Materials, Harbin Institute of Technology (Shenzhen), Shenzhen 518055, China

**Keywords:** solder, intermetallic, TEM, CoSn_3_ nanoparticles, chemical reduction

## Abstract

At present, composite solder pastes are getting a lot of attention, especially composite Sn based solders reinforced by nanoparticles. Indeed, CoSn_3_ is a strong nucleating agent of Sn crystal, which has potential application value in the field of electronic packaging. However, there is no reliable synthetic path for CoSn_3_ nanoparticles at present. In this article, a chemical synthesis method for CoSn_3_ nanoparticles is developed. Here, CoCl_2_ and SnCl_2_ are reduced by NaHB_4_ in triethylene glycol (TEG), dispersed by ultrasonics, and heated to 350 °C in a tube furnace for growth. The CoSn_3_ nanoparticles with a diameter of about 150 nm are obtained by heating at 350 °C for 10 min. The CoSn_3_ nanoparticles undergo a step reaction in the process of synthesis and go through different stages of merging and annexation during their growth. The crystal growth behavior and the process of orientation change during the nucleation and growth of CoSn_3_ nanoparticles are studied, especially the two growth mechanisms, namely OU (orientation unified) and OA (orientation attached). By mixing CoSn_3_ nanoparticles with SAC305, we obtain a kind of strengthened composite soldering paste. There are obvious six-fold cyclic twins in the joints made by this soldering paste.

## 1. Introduction

Three-dimensional integrated circuit (3D-IC) technology has emerged as the most promising new technology for the future realization of “more Moore’s law”. In the development of 3D-IC technology [1], copper pillar bumps are becoming the main choice for electronic package interconnects with a pitch of 100 μm or less owing to their good electrical and thermal conductivity [2,3]. Furthermore, Sn-based solder interconnects are the most used bonding technology owing to their low cost and high throughput, while the increase in packaging density means a decrease in the number of Sn grains in the copper pillar bumps. The orientation anisotropy of *β*-Sn due to its body-centered tetragonal (BCT) crystal structure (a [100] = b [010] = 5.83 Å and c [001] = 3.18 Å) leads to strong anisotropy in TM and EM behavior and causes failure of electronic devices at high current densities [4,5].

To meet the above challenges, composite solder paste is an important development direction. Composite solder refers to directly adding reinforcing particles into Sn based solder paste, and then mechanically mixing them with solder paste for a long time to prepare a composite solder paste, which is directly used for surface assembly process. By introducing the strengthening phase, the solder with a stable microstructure and uniform deformation can be obtained. Another important feature of this strengthening mechanism is that it will not change the melting point, wettability, and other process characteristics of the original solder. For example, CoSn_3_ nanoparticles are added to Sn based solder paste. The CoSn_3_ can be added as heterogeneous nucleating agents to design and produce solder joints with fine grains.

**Figure 1:**
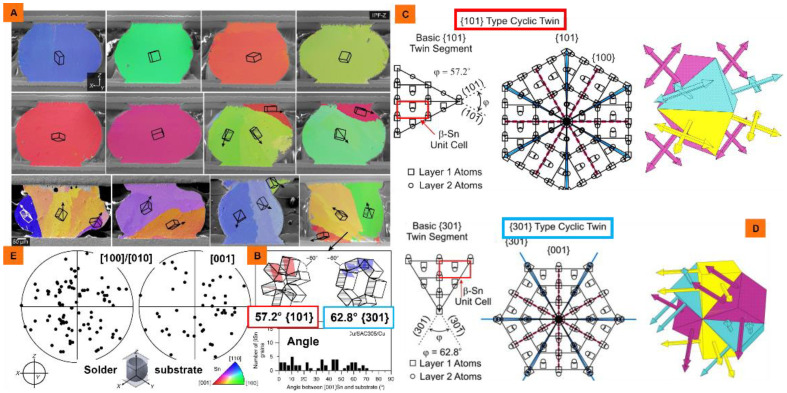
{101} (**A**) or {301} (**B**) type twins induced by CoSn_3_ substrate, rotating 57.2° and 62.8° around [100], respectively (**C**) Projection view of Sn lattice onto the (0 1 0) plane, showing a basic {1 0 1} twin segment and a {1 0 1} cyclic twin nucleus. (**D**) Projection view of Sn lattice onto the (0 1 0) plane, showing a basic {3 0 1} twin segment and a {3 0 1} cyclic twin nucleus (**E**) <100> and <001> pole figures showing the highly variable *β*Sn grain orientations (adapted from [6,7,10]).

The CoSn_3_ is a relatively simple lattice match in terms of orientation relationships (ORs) between Sn crystals and CoSn_3_ because it has a crystal structure like that of Sn crystals. Therefore, CoSn_3_ can be used as a more potent heterogeneous nucleating agent, and the orientation of *β*-Sn grains that undergo nucleation events is unchanged, since CoSn_3_ mostly inherits the orientation from CoSn_3_, with the inheritance relationship of OR: (100)Sn||(100)CoSn_3_ versus [001]Sn||[001]CoSn_3_ (Figure 1).Notably, there is a lack of methods for the synthesis of CoSn_3_ nanoparticles [6,7].

Cobalt is a paradoxically fast diffusing element in Sn, and the preparation of CoSn_3_ nanoparticles in past studies usually involves the reduction reaction of sodium borohydride and precursors in a hydrothermal reactor for about 72 h [8,9], which has a long reaction time, low reaction efficiency, and poor safety. For example, Cai [8] et al. obtained a single CoSn_3_ phase by using ethylene glycol as the solvent, CoCl_2_-6H_2_O and SnCl_2_ as the precursor solution, and NaHB_4_ as the reducing agent, and reacting at 250 °C for 72 h in a reactor.

In this study, CoSn_3_ nanoparticles ae prepared by using triethylene glycol (TEG) as the solvent and ethanol solution of CoCl_2_ and SnCl_2_ under the action of the reductant NaHB_4_ and ultrasound, and a method for the synthesis of CoSn_3_ nanoparticles by ultrasonic reduction and tube furnace growth was developed. Nanoparticles with different heating times were employed. The research focuses on the crystal growth behavior and orientation mechanism during the nucleation and growth of CoSn_3_ nanoparticles, especially the observation of two growth mechanisms, namely orientation unification (OU) and orientation attachment (OA).

## 2. Materials and Methods

The CoSn_3_ nanoparticles was successfully prepared via the sodium borohydride reduction method. The precursors used for the preparation of CoSn_3_ nanoparticles were CoCl_2_ and SnCl_2_, and the detailed specifications of other chemical reagents, such as the organic carriers used for the preparation of nanoparticles, are shown in Table 1.

**Figure 2:**
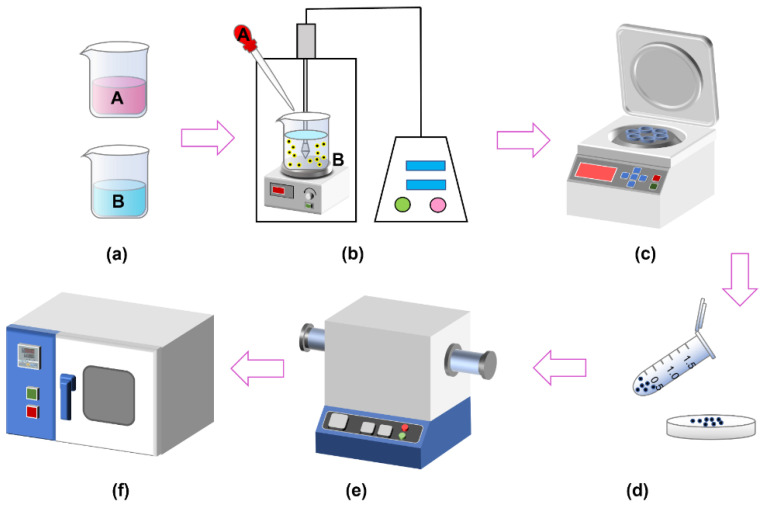
Flow chart for the preparation of CoSn_3_ Nps.(**a**) Prepare solution A and solution B (**b**) Reduction in ultrasonic environment (**c**) The product of reduction reaction is obtained by centrifugation after deionized water cleaning (**d**) The product is transferred to the crucible and ethylene glycol is added.(**e**) Tube furnace heating (**f**) After acid pickling with 15% dilute salt, the products are washed with deionized water and ethanol respectively, and then dried.

**Solution A** was obtained by adding anhydrous cobalt chloride and stannous chloride dihydrate to 50 mL of ethylene glycol solution. Triethylene glycol (TEG) and ethanol were mixed in a volume ratio of 1:5 to obtain 300 mL of Solution B. Dimethyldiallylammonium chloride was added to adjust the pH of the solution to 6.0 and 0.1 g of polyvinylpyrrolidone (PVP) was added. Solution B was placed in the ultrasonic disperser, accompanied by magnetic stirring at a stirring rate of 200 rpm/min, ultrasonic power of 480 W, frequency of 20 KHz. We added Solution A to Solution B drop by drop using a rubber-tipped dropper (Figure 2a,b).

**Figure 3:**
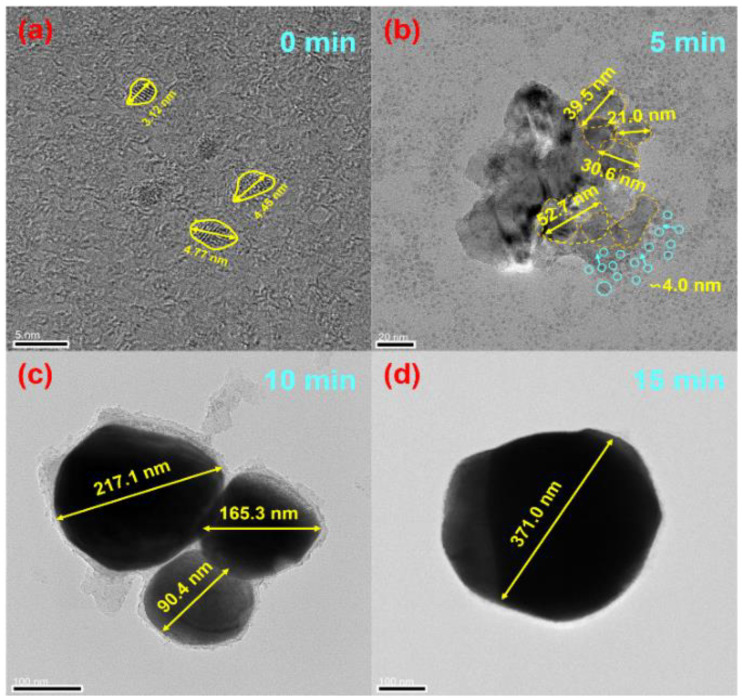
TEM images of CoSn_3_ Nps. (**a**) Heating at 350 °C for 0 min; (**b**) heating at 350 °C for 5 min; (**c**) heating at 350 °C for 10 min; (**d**) heating at 350 °C for 15 min.

The products after reduction with ultrasonic were mainly a series of Co–Sn IMCS nanoparticles. The products was obtained by centrifugation, added to ethylene glycol, and placed in a tube furnace at 350 °C with an atmosphere of N_2_ + H_2_ (Figure 2c–e). CoSn_3_ nanoparticles with a diameter of 100–150 nm can be obtained by heating for about 10 min. The heating time in the tube furnace determines the diameter of the nanoparticles (Figure 3).

The samples were removed from the tube furnace at 5 min of heating and 10 min of heating, respectively, and cleaned using diluted hydrochloric acid to obtain nanoparticles (Figure 2f). The method of ultrasonic reduction–heating growth can rapidly prepare CoSn_3_ intermetallic compound nanoparticles, with fast reaction speed and high safety, without the need for a reactor necessary for hydrothermal synthesis. All the preparation is carried out under atmospheric pressure, without high pressure, ultra-high temperature, and other harsh conditions. The reaction by-products are mainly salts, which can be removed by deionized water cleaning.

**Figure 4:**
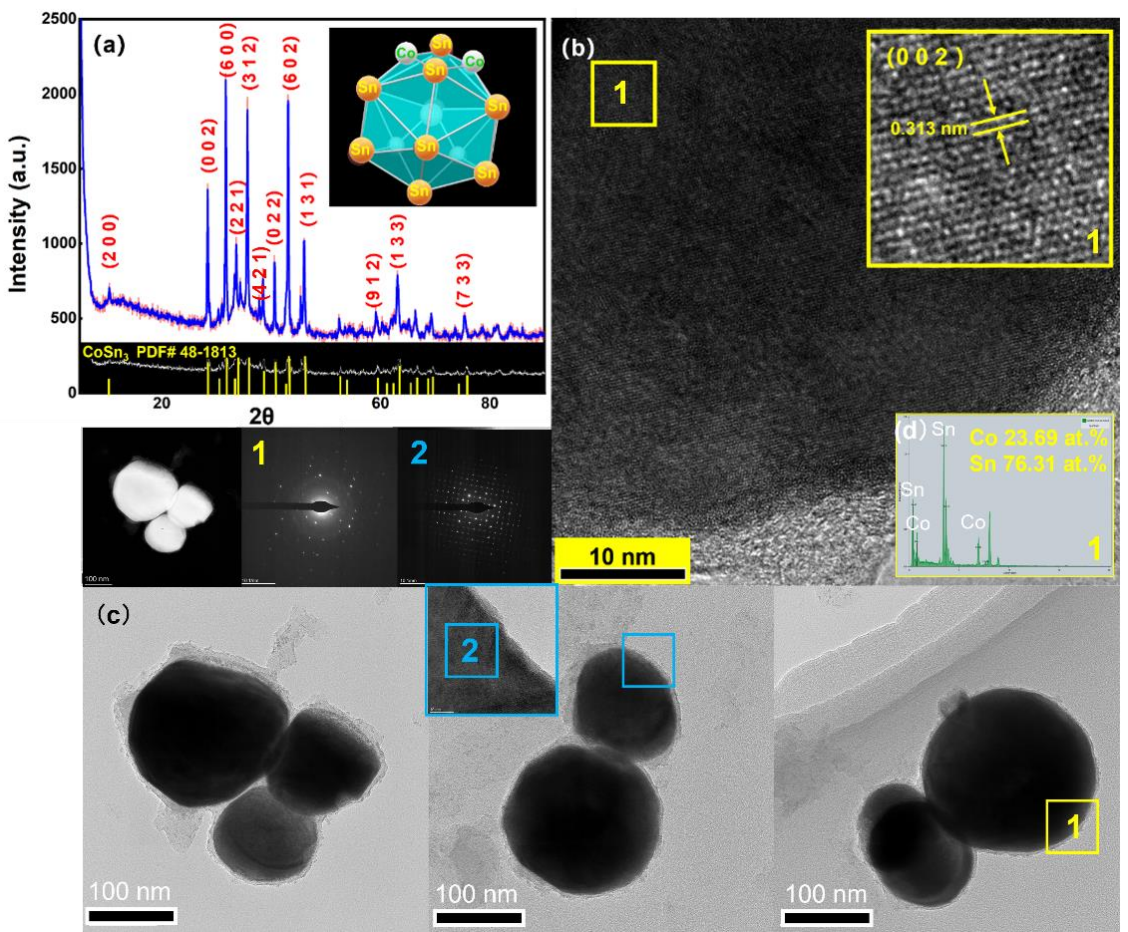
(**a**) XRD patterns of CoSn_3_ NPs; (**b**) the high-resolution image of CoSn_3_ NPs; (**c**) TEM images of CoSn_3_ NPs. The number represents the sampling area. (**d**) EDX Result of location 1 in (**b**).

**Figure 5:**
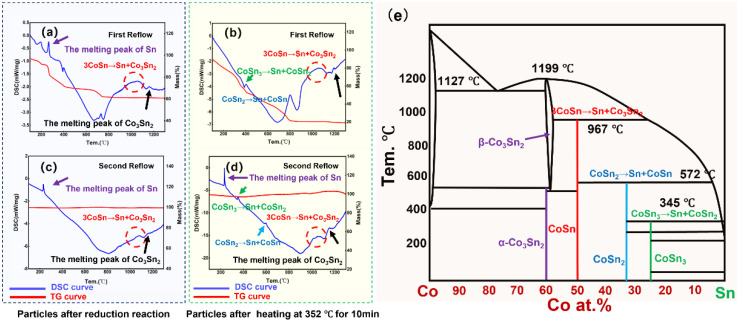
Results of DSC-TG experiments. (**a**) First DSC-TG experiment of pellets obtained by drying samples after reduction sonication; (**b**) first DSC-TG experiment of samples heated in a tube furnace for 15 min; (**c**) second DSC-TG experiment of pellets obtained by drying samples after reduction sonication; (**d**) second DSC-TG experiment of pellets obtained by drying samples after reduction sonication; (**e**) Co–Sn binary phase diagram.

The microstructure and elemental distribution of nanoparticles were characterized by transmission electron microscopy (TEM, FEI^®^ Talos F200X G2, Hillsboro, OR, USA) with energy dispersive spectrometry (EDS) and high angle annular dark field (HAADF) imaging. The dispersant selected was ethylene glycol, and the ultrasound was performed for 3 min before observation. The bright-field image (BF), high-angle annular dark-field image (HADDF), selected area electron diffraction (SAED), and high-resolution image (HRTEM) were also selected to observe the tissue morphology and perform phase identification. Furthermore, X-ray diffraction (XRD) patterns were used to analyze the crystallinity of the prepared nanoparticles with the equipment manufactured by the Rigaku Corporation^®^ (Tokyo, Japan), model D/max 2500; the Scanning range was 10°–90°, while the scanning speed was 10 deg/min (Figure 4). The XRD and DSC experiments were performed to reveal the reactions occurring during the synthesis of CoSn_3_ nanoparticles (*NPs*). The differential scanning calorimetry (DSC) was used to test the performance of nanoparticles subjected to heat, and the STA-449F5 used in this experiment was from NETZSCH^®^, Selb, Germany. The experimental parameters were as follows: nitrogen atmosphere, heating rate 10 °C/min, and heating range 25~1300 °C. The samples in the DSC experiment were heated before and after heating at 350 °C (Figure 5a,b), respectively, and each set of samples was heated to 1300 °C, cooled and heated again to 1300 °C to exclude the interference of organic residues (Figure 5c,d). 

## 3. Result and Discussion

The XRD of the CoSn_3_ NPs shows that the growth of the grains is along isotropic lines, and the crystallinity is good. The diameter of the synthesized nanoparticles is about 100 nm, and the crystal structure belongs to the tetragonal crystal system (I4_1_cd, Figure 4).

### 3.1. Reductive Reaction

The nanoparticles show complex heat absorption and exothermic changes in the thermal environment, revealing that the CoSn_3_ particles may have complex degradation mechanisms at high temperatures, which need to be further investigated. The difference between the results of the two samples is that the TG curve (red curve) in (a) (b) shows a decreasing trend followed by a smooth trend, resulting from the volatilization and pyrolysis of organic residues (c), while the two curves in (d) have identical heat absorption peaks, indicating that the sample that did not crystallize during the heating of the first experiment. It can be seen that (a) also completed crystallization. Comparison of (b) (d) shows that the melting peak of tin, which was not present during the first heating, appeared during the second heating, indicating that CoSn_3_ degrades into CoSn_2_, CoSn, and Co_3_Sn_2_ during the first heating and precipitates Sn. Thus, the phase change that occurs during the heating of nanoparticles is as follows. This phenomenon of thermal step degradation reveals the existence of step reactions in the synthesis of nanoparticles.

CoSn_3_→Sn + CoSn_2_

CoSn_2_→Sn + CoSn

3CoSn→Sn + Co_3_Sn_2_

**Figure 6:**
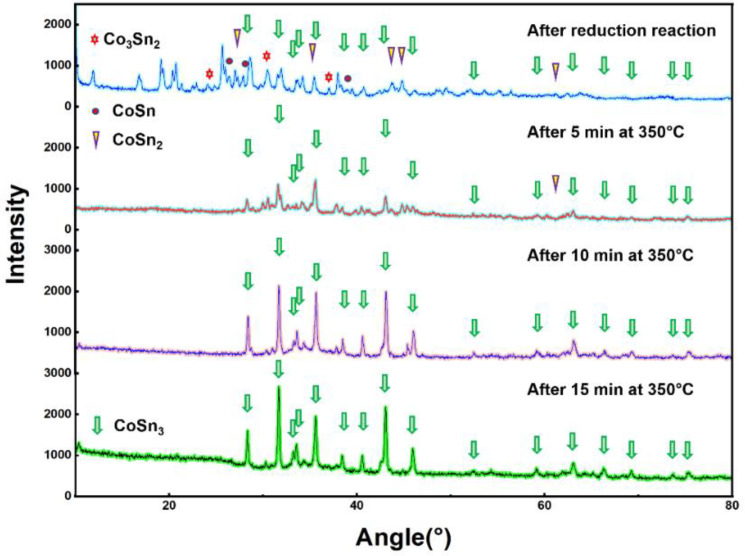
XRD scanning pattern after heating in a tube furnace for 0 min, 5 min, 10 min, and 15 min.

After the reduction reaction, XRD tests are performed on the solid precipitation products, at 350 °C for 5 min, 350 °C for 10 min, and 350 °C for 15 min for the samples, respectively (Figure 6). The results showed that the products after the reduction reaction were CoSn, CoSn_2_, and Co_3_Sn_2_, and that only CoSn_3_ nanoparticles remained after heating at 350 °C.

To summarize, Co^2+^ and Sn^2+^ are first reduced to particles of Co and Sn by NaBH_4_. The reaction equation is shown as follows:

CoCl_2_ + 2NaBH_4_ = Co + 2BH_3_ + 2NaCl + H_2_

SnCl_2_ 2H_2_O + 2NaBH_4_ = Sn + 2BH_3_ + 2NaCl + H_2_ + 2H_2_O

**Figure 7:**
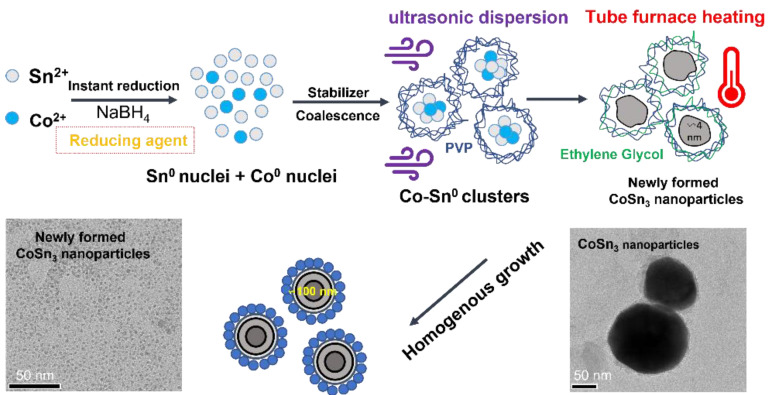
Schematic diagram of the formation of CoSn_3_ nanoparticles.

**Figure 8:**
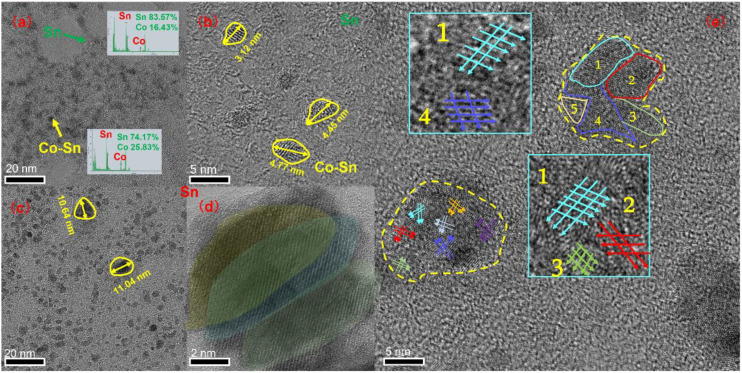
CoSn_3_ nanoparticles after heating for 0 min at 350 °C. (**a**) TEM image; (**b**) TEM image; (**c**) TEM image; (**d**) Mohr pattern; (**e**) orientation unification. The number represents the sampling area.

During the reaction, the reaction that produces Sn particles will proceed preferentially because the standard electrode potential of Sn^2+^/Sn in solution is −0.14 V, which is higher than the standard electrode potential of Co^2+^/Co (−0.28 V). Cobalt is a fast-diffusing element in Sn because Sn is generated before Co during the reaction, so simple Co–Sn compounds are distributed in Sn after the reduction reaction (Figure 7 and Figure 8). The energy provided at room temperature is far from the activation energy required for the reaction between Co and Sn to form CoSn_3_, so external input energy is required to synthesize CoSn_3_ NPs. The tube furnace can provide energy quickly. 

A mixture containing Sn and Co is heated and the more reactive Co and Sn synthesize CoSn_3_ NPs. The possible reactions occurring in the process are as follows:

3Co + 2Sn = Co_3_Sn_2_

Co_3_Sn_2_ + Sn = 3CoSn

CoSn + Sn = CoSn_2_

CoSn_2_ + Sn = CoSn_3_

### 3.2. Growth

**Figure 9:**
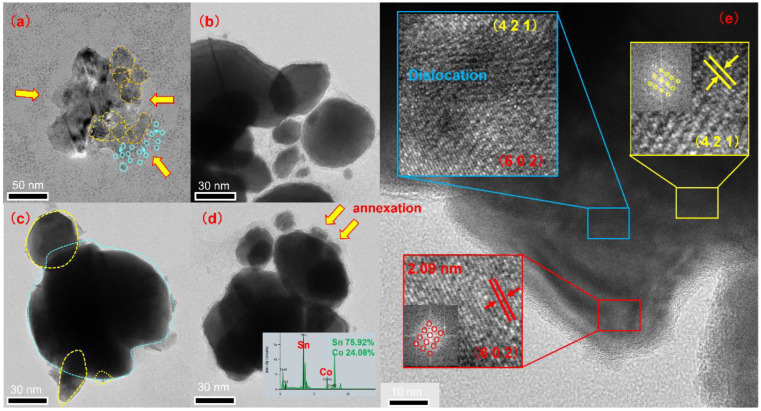
CoSn_3_ nanoparticles after heating for 5 min 350 °C. (**a**) TEM image; (**b**–**d**) large particles engulfing small ones; (**e**) orientation attachment in the process of nanoparticles merging.

The nucleation and growth mechanism of CoSn_3_ nanoparticles is investigated by transmission electron microscopy (TEM). Here, “orientation homogeneity (OU)” and “orientation attachment (OA)” phenomena are observed during the incorporation and engulfment of CoSn3 nanoparticles. Prior to heating, the Co–Sn IMC NPs in Figure 8 have a relatively uniform particle size with an average diameter of ∼4 nm, which increases to an average diameter of ∼30 nm when heated at 350 °C for 5 min (Figure 9), with a more inhomogeneous Gaussian-like distribution. The above behavior follows Frenkel’s theory, which describes the auto-diffusion of solids between crystals in contact, driven by the reversion of surface energy [10,11,12]. The equation is as follows:

$$\frac{\pi }{3}a^{3}(2+\cos\theta -\cos^{3}\theta )=\frac{4\pi }{3}a_{0}^{3}$$

where *a* is the radius of the spherical portion of the particles during sintering and is the angle between the line through the centers of the two spheres and the line between the center of the spheres and the edge of the necking region. After time *t*, the radius becomes as follows:

$$a(t)=a_{0}{\left(\frac{4}{{\left(1+\cos\theta \right)}^{2}(2-\cos\theta )}\right)}^{\frac{1}{3}}$$

where *a*_0_ is the original particle size. The necking area of adjacent particle spheres increases with increasing sintering time *t*. To coarsen to a single large particle, the time required is as follows:

$$t=\frac{4}{3}\frac{\eta a_{0}}{\sigma }=\frac{4}{3}\frac{a_{0}}{\sigma }\frac{kTa^{3}}{D\delta_{}^{3}})$$

where *T* is the sintering temperature, *D* is the diffusivity, which is the atomic distance in the crystal, and *k* is the Boltzmann constant. Here, *D* is related to the particle size and temperature, as follows:

$$D=D_{0}\exp\left\{\frac{-E(\infty )}{RT}\exp\left[\frac{-2S(\infty )}{3R}\frac{1}{r/r_{0}-1}\right]\right\}$$

where *E*(∞) is the diffusion activation energy of the bulk material, *S*(∞) is the entropy of melting of the bulk, *D*_0_ is the diffusion rate of the bulk, *r* is the particle radius, and *R* is the gas constant. Therefore, according to the above equation, decreasing the particle size, and increasing the temperature and sintering time can promote the agglomeration of NPs, which matches well with the above experimental phenomena.

However, it is found in the experiment that the growth rate of the particles is not as fast in the first 5 min (∼4 nm–20 nm) as in the last 5 min (∼30 nm–150 nm) under heating at 350 °C. After 5 min heating (Figure 9), the small particles around the large particles are smaller than 10 nm (diameter). The large particles in the middle also range from ∼30 nm to 35 nm. A similar phenomenon occurs in other monitored regions where larger size differences lead to faster coarsening. The main reason can be explained by the asymmetric spherical necking model. During the sintering process, the interfacial energy can be described as follows:

$$E=\gamma_{S}A_{S}+\gamma_{b}A_{b}$$

where *γ_s_* is the surface energy, *γ_b_* is the boundary energy, as is the surface area, and *A_b_* is the boundary area. For spheres with different volumes, the normalized energy is reduced to [13], as follows:

$$\Delta E*=2^{-2/3}\left\{{\left(1-V_{\beta }\right)}^{2/3}+V_{\beta }{}^{2/3}\right\}-1$$

Combining two particles with the same size is the least energy preferable, and the larger the size difference, the more energy will be released by coarsening, which leads to faster sintering. In addition to this, due to the high density of grain boundaries brought about by the nanostructure, the scattering ability of phonons greatly exceeds the scattering of phonons by carriers, and the high thermal conductivity of the particles, so that the growth rate of the particles is greatly accelerated in the later stages of heating.

In Figure 8d, the overlapping area of two CoSn_3_ NPs is more than half of their cross-section. During the unification of these two particle orientations, a moiré stripe pattern appears. With continued heating, the density of the moiré stripe pattern decreases, indicating a decrease in orientation errors due to the transformation of the crystal structure (Figure 9e). This lasts until the two particles merge with each other in a uniform orientation and become rounder and larger particles. This phenomenon is called orientation unification (OU) [14,15].

If the contact area of two particles is less than 50% of the cross-sectional area of the smaller particle and the difference in original orientation is large, orientation attachment (OA) may occur, as shown in Figure 8, where the central particle increases in size by consuming the surrounding smaller particles. As heating continues, the dislocations gradually disappear, eventually creating a more uniform contrast. The CoSn_3_ NPs with different orientations (Figure 9) can merge with each other. However, there is not enough time and energy to adjust the crystal structure, so dislocations occur at the interface. With further coarsening, the dispersed distribution of dislocations and dislocations produced by the merging of multiple particles is normalized and merged into several major dislocations. If two neighboring particles have the same orientation, they can easily merge with each other without creating any defects; however, when their original orientations are different, dislocations may arise at the interface. In Figure 9e, when the large particles complete the annexation of small particles, but the orientation of small particles is still retained, there are two different orientations at the edge of a completed nanoparticle. Furthermore, there are dislocations between the two oriented regions [12,13,14].

In the early stage of nanoparticle growth, the merging of particles of a similar size is the main phenomenon, and the phenomenon of “orientation unification” is found. At the later stage of particle growth, the phenomenon of “orientation adhesion” is found, as the larger particles engulf the smaller ones.

### 3.3. Application of CoSn_3_ Nanoparticles in Electronic Packaging

**Figure 10:**
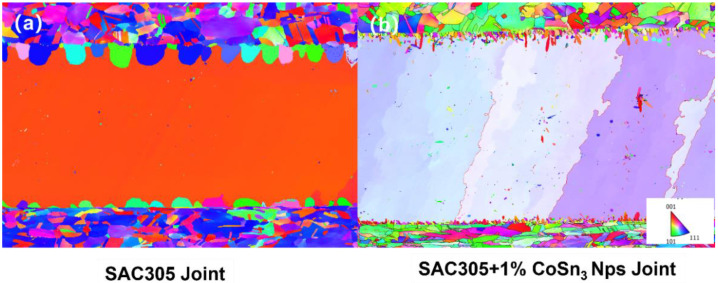
EBSD analysis results. (**a**) SAC305 joint; (**b**) SAC305 + 1 wt.% CoSn_3_ nanoparticles.

The synthesized nanoparticles are added to SAC305 solder paste with the addition of 1% mass ratio, and the joints are obtained by pressure-less reflow at 230 °C. Six-fold cyclic twinning of Sn crystals can be obviously induced to achieve the purpose of refining grains (Figure 10).

The *β*-Sn crystal is more complex than most metals and has the closest close packed plane (010), which includes the close packed direction [100] and the sub-close packed direction [001]. Different IMCs have different effects on Sn crystal nucleation. For highly effective heterogeneous nucleating agents (such as CoSn_3_), the orientation relationships (ORs) between Sn crystals and IMC are relatively simple lattice matching (Figure 1). The ORs of heterogeneous nucleating agents (Cu_6_Sn_5_, Ag_3_Sn, and Ni_3_Sn_4_) with low potency and have more complex lattice matching. The *β*-Sn solidification is very complicated in the process of nucleation with the help of heterogeneous IMC; generally, the twin axis of [100] Sn will occur along the direction of the lowest linear atomic segregation (the lowest segregation) on the CoSn_3_, and form interrelated cyclic twins, which are related by sharing the [100] axis. Therefore, there are multiple heterogeneous nucleation sites on the IMC plane *β*-, and the Sn grain direction is connected by the ring twin structure. All grains are related by rotating 60 ° around the common [100] axis. Therefore, although the addition of CoSn_3_ NPs can realize the occurrence of multiple nucleation events in solder joints, these grains are still interrelated. The mechanism and model here still need to be further studied.

## 4. Conclusions

The following conclusions may be drawn:

(1) CoSn_3_ nanoparticles are successfully prepared via a sodium borohydride reduction method. The precursors used for the preparation of CoSn_3_ nanoparticles are CoCl_2_ and SnCl_2_, and the reducing agent is NaBH_4_. The reduction products are heated in a tube furnace at 350 °C for 10 min (N_2_ + H_2_) to obtain CoSn_3_ nanoparticles with a size of about 150 nm;
(2) CoSn_3_ nanoparticles undergo a step reaction in the process of synthesis, as follows: Co_3_Sn_2_ -CoSn-CoSn_2_-CoSn_3_; AND CoSn_3_ nanoparticles undergo degradation by heat in the order of CoSn_3_-CoSn_2_-CoSn-Co_3_Sn_2_, precipitating Sn;
(3) During the growth of CoSn_3_ nanoparticles by heat, the larger the particle diameter, the faster the growth. In the early stage of nanoparticles growth, the merging of particles of a similar size is the main phenomenon, and the phenomenon of “orientation unification” is found. At the later stage of particle growth, the phenomenon of “orientation adhesion” is found, as the larger particles engulf the smaller ones.

## Figures and Tables

**Table 1:** Required raw materials and chemical reagents.

Sodium Borohydride	NaBH_4_	AR	Aladdin^®^
Cobalt chloride	CoCl_2_	AR	Aladdin^®^
Stannous chloride	SnCl_2_·2H_2_O	AR	Aladdin^®^
Triethylene glycol	C_6_H_14_O_4_	AR	Aladdin^®^
Polyethylene glycol	HO(CH_2_CH_2_O)_n_H	AR	Aladdin^®^
Ethylene glycol	(CH_2_OH)_2_	AR	Aladdin^®^
Polyvinylpyrrolidone	(C_6_H_9_NO)_n_	AR	Aladdin^®^
Anhydrous ethanol	C_2_H_6_O	AR	Aladdin^®^
Dimethyldiallylammonium chloride	C_8_H_16_NCl	AR	Aladdin^®^
OM338PT SAC305	Sn-3.0Ag-0.5Cu	∽90%	ALPHA^®^
